# Molecular and immunological characterisation of peanut ingredients for double blind placebo controlled food challenge (DBPCFC)

**DOI:** 10.1186/2045-7022-3-S3-P176

**Published:** 2013-07-25

**Authors:** P Johnson, A Balasundaram, A Taekema, N Rigby, C Mills

**Affiliations:** 1 Institute of inflammation and Repair, University of Manchester, Manchester, UK; 2 Wageningen University, Wageningen, the Netherlands; 3 Institute of Food Research, Norwich, UK

## Background

Double blind placebo controlled food challenge (DBPCFC) is the gold standard for food allergy diagnosis and outcome measure. However, one difficulty in performing DBPCFC is the lack of well-characterised and validated sources of allergens for incurrence into the challenge food vehicle. Here, we discuss the requirements for this characterisation using the peanut flour used during the EuroPrevall project and in ongoing studies.

## Methods

2-D PAGE and mass spectrometry (Thermo Fisher Orbitrap Elite) was used to provide a molecular characterisation of allergens in peanut flour. Relative quantitation was used to determine batch-to-batch variation in allergen content. Immunoreactivity of the peanut flour material was assessed using rabbit polyclonal antisera raised to purified peanut allergens.

## Results

2D PAGE profiles of peanut flour showed little variation between batches. Tandem mass spectrometry showed several isoforms of each of the major peanut allergens (Ara h 1, 2, 3, 4, and 6) as well as less abundant allergens (Ara h 7, 8, 9 and 10) were present in the flour samples. Label-free quantitation was able to demonstrate equivalent quantities of the allergens and, in some cases, their individual sequence isoforms in different batches of peanut flour. Immunoblotting showed that Ara h 2/6 was unaffected by thermal processing in the peanut ingredient but Ara h 1 was rendered undetectable.

## Conclusion

Molecular profiling methods have shown that a peanut flour ingredient used for inclusion in oral food challenges is consistent across several batches. The combination of physicochemical characterisation of allergens in peanut ingredients, coupled with an assessment of their immunological reactivity provide a good basis for quality control and assuring that they are similar with regard to allergen content.

## Disclosure of interest

None declared.

